# Morbidity and Mortality Outcomes After Cytoreductive Surgery with Hyperthermic Intraperitoneal Chemotherapy for Treatment of Ovarian Cancer

**DOI:** 10.3390/jcm14051782

**Published:** 2025-03-06

**Authors:** Migang Kim, Yong Jae Lee, Ki Eun Seon, Sunghoon Kim, Chan Lee, Hyun Park, Min Chul Choi, Jung-Yun Lee

**Affiliations:** 1 Comprehensive Gynecologic Cancer Center, CHA Bundang Medical Center, School of Medicine, CHA University, Seongnam 13520, Republic of Korea; mgkim87@chamc.co.kr (M.K.); chanoncology@chamc.co.kr (C.L.); p06162006@cha.ac.kr (H.P.); 2 Department of Obstetrics and Gynecology, Institute of Women’s Life Medical Science, Yonsei University College of Medicine, Seoul 06229, Republic of Korea; svass@yuhs.ac (Y.J.L.); kesunova@gmail.com (K.E.S.); shkim70@yuhs.ac (S.K.)

**Keywords:** hyperthermic intraperitoneal chemotherapy, ovarian cancer, postoperative complications, morbidity

## Abstract

**Background/Objectives**: Hyperthermic intraperitoneal chemotherapy (HIPEC) with cytoreductive surgery (CRS) has been reported to improve survival in patients with peritoneal carcinomatosis. This study aimed to investigate the morbidity and mortality rates of CRS with HIPEC in patients with ovarian cancers. **Methods**: We retrospectively reviewed the medical records of patients who underwent CRS with HIPEC for ovarian cancer from January 2013 to July 2021 at two tertiary institutions. The morbidities and mortalities that occurred within 30 days after HIPEC and the clinical and operative factors related to morbidities were investigated. **Results**: A total of 155 procedures in 151 patients were included in this study. The median age was 55 years and the median score of the peritoneal carcinomatosis index was eight points. Morbidities of grade ≥3 within 30 days of HIPEC occurred in 18 patients (11.6%). The most common severe morbidity was wound infection (3.2%), followed by pleural effusion (1.9%) and postoperative hemorrhage (1.9%). Within the 30-day postoperative period, there were no reported mortality cases. There were statistical differences in age, length of stay, peritoneal carcinomatosis index, bowel resection, operation time, and completeness of cytoreduction between the patients and severe morbidity. However, in the multivariate logistic analysis, none of the factors showed a statistically significant relationship with the occurrence of severe morbidity. **Conclusions**: The morbidity and mortality rates of CRS with HIPEC in gynecologic cancer patients were relatively low compared to those in previous reports. Further studies about the possible risk factors are needed.

## 1. Introduction

Peritoneal carcinomatosis is a common form of metastasis in ovarian cancers. In approximately 60% of cases, peritoneal metastases are reported with ovarian cancer at the time of diagnosis [1,2]. To maximize cytotoxic effects, intraperitoneal (IP) chemotherapy was introduced, allowing the direct administration of anticancer drugs into the peritoneal cavity at a high concentration. The Gynecologic Oncology Group conducted a GOG-172 trial, comparing IP chemotherapy with intravenous chemotherapy in patients with optimally debulked ovarian cancer. In that study, IP chemotherapy showed improved overall survival and progression-free survival compared to conventional intravenous chemotherapy [3]. Despite these benefits, the widespread adoption of IP chemotherapy was limited due to several reported adverse events, such as pain and gastrointestinal and catheter-related problems, in previous studies [3,4,5].

Hyperthermic intraperitoneal chemotherapy (HIPEC) has been used as another type of intraperitoneal anticancer drug administration. HIPEC is a surgical method of the perfusion of heated chemotherapeutic agents into the abdominal cavity for 60–90 min, following optimal cytoreductive surgery (CRS). It has been used for the treatment of peritoneal carcinomatosis in gynecological and gastrointestinal cancers. The synergy of the hyperthermic effect on the tumor and direct delivery of the drug into the peritoneum resulted in longer overall survival and progression-free survival than when only CRS was performed in previous studies [6,7,8].

The number of ovarian cancer patients undergoing HIPEC increased in the United States after the publication of the study by van Driel in 2018, which demonstrated the efficacy of HIPEC with interval debulking surgery (IDS). The estimated percentage of HIPEC with IDS for ovarian cancer rose by 0.08% per month, reaching a peak of 3%. Notably, 97% cases of HIPEC performed between 2016 and 2020 were conducted after the van Driel’s report in 2018 [9]. However, physicians may have concerns about postoperative morbidity following HIPEC due to the prolonged operation time and the use of additional anticancer drugs during surgery. The previous retrospective studies reported a broad rate of severe morbidity of HIPEC of 8.6–35.7% [10,11,12,13,14,15]. There were few studies that focused on postoperative morbidity and mortality with HIPEC in the field of ovarian cancer, and the majority of the studies were limited to gastrointestinal cancers. The morbidity rate and profile could differ according to the tumor origin, the extent of surgery, the timing of surgery, and the type of anticancer drug used. Thus, in this study, we investigated the rates of postoperative morbidity and mortality and factors related to the occurrence of morbidity when HIPEC was performed after CRS in ovarian cancer.

## 2. Materials and Methods

### 2.1. Patients

**Figure 1:**
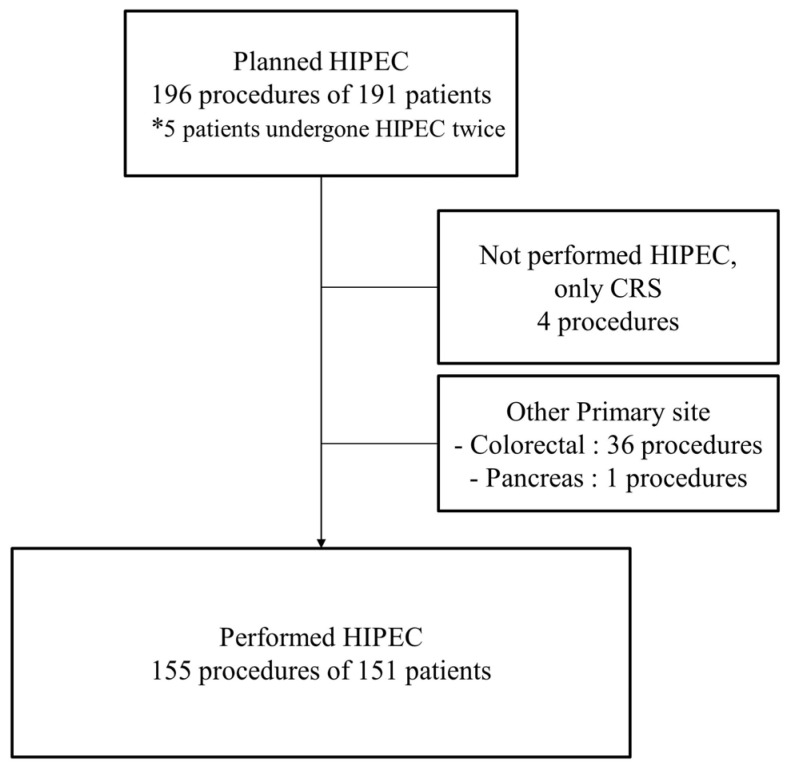
Flow chart of patient selection. HIPEC, hyperthermic intraperitoneal chemotherapy; CRS, cytoreductive surgery. * In this study, patients who had undergone HIPEC twice were included.

This retrospective study was conducted at two tertiary cancer centers in Korea. We conducted a review of the medical records for 196 procedures performed on 191 patients scheduled for HIPEC with CRS between January 2013 and July 2021. The inclusion criteria were as follows: (1) patients pathologically diagnosed with primary ovarian, peritoneal, and fallopian tubal cancer; and (2) patients who underwent HIPEC with CRS regardless of the timing of surgery, such as primary debulking surgery (PDS), IDS, and secondary or tertiary CRS. Among these patients, those who underwent CRS but did not undergo HIPEC were excluded from the study. (Figure 1) This study was approved by the Institutional Review Board (CHAMC 2021-08-014).

### 2.2. Procedures of Cytoreductive Surgery and Hyperthermic Intraperitoneal Chemotherapy

CRS and HIPEC were performed according to the institutional protocol. All sites suspected of metastasis were resected during the CRS, including the uterus and ovaries. Parietal peritonectomy and greater and lesser omentectomy were also performed. The tumor burden was determined based on the peritoneal carcinomatosis index (PCI) score. Residual tumors were classified intraoperatively using the completeness of the cytoreduction (CC) system [16]. CC-0 indicates no residual tumors and CC-1 indicates a residual tumor less than 2.5 mm. CC-2 and CC-3 indicate a residual tumor between 2.5 mm and 2.5 cm, and larger than 2.5 cm, respectively.

HIPEC was performed immediately after CRS with an open technique with a laparotomy cytoreduction or closed technique after a laparoscopic cytoreduction, depending on the institution and surgical preference. Two inflow and two outflow drainage tubes were placed sub-diaphragmatically and in the pelvic cavity. The abdominal cavity was lavaged with 2 L of normal saline before HIPEC. Then, 2500–3500 mL of the diluted normal saline or diluted 1.5% dextrose solution for peritoneal dialysis with chemotherapy agent was circulated with an extracorporeal circulation device and hyperthermic infusion pump (Belmont Instrument Corporation, Billerica, MA, USA), for 60–90 min with a flow rate of 1000 mL/min after 10–20 min of preheating with normal saline. The chemotherapeutic agents used were paclitaxel (175 mg/m^2^) or cisplatin (100 mg/m^2^). The intra-abdominal target temperature was 41.5–42.5 °C and it was measured using two intraperitoneal temperature probes. To prevent nephrotoxicity in patients receiving HIPEC with cisplatin, sodium thiosulfate was administered concurrently with cisplatin. A bolus of 9 g sodium thiosulfate per body surface area was given at the same time as HIPEC, followed by an additional intravenous infusion of 12 g per body surface area over six hours. After HIPEC, the abdominal cavity was lavaged three times with 2 L of normal saline.

### 2.3. Data and Statistical Analysis

We collected information on relevant clinical data including the surgical complexity score, postoperative morbidity, and mortality rates. The surgical complexity score (SCS) was calculated based on the Aletti scoring system [17,18]. The SCS was stratified into three groups: low, intermediate, and high. Safety analyses encompassed 30-day surgical morbidity and mortality, as well as treatment-related adverse events according to the Memorial Sloan Kettering Cancer Center (MSKCC) surgical secondary event (SSE) system, a modified Clavien–Dindo classification [19]. Grade 3–5 morbidities were defined as severe morbidity. In addition, in cases where several unrelated morbidities occurred, each was individually graded, and the overall morbidity rate was calculated based on the most severe degree.

Mortality was identified as death occurring within 30 days postoperatively, regardless of the cause of death. We additionally collected data related to morbidity and mortality between 31 to 90 days after surgery, as well as re-hospitalization or re-operation rates.

Continuous variables were presented as medians with ranges, and categorical variables were presented as frequencies and percentages. To compare the differences in clinical factors according to the occurrence of severe morbidities, the Mann–Whitney U test was used for continuous variables. All categorical variables were analyzed using Pearson’s chi-squared and Fisher’s exact tests. Univariate logistic regression analysis was performed in all study populations with independent variables to explore factors related to 30-day morbidities and confirm their influence. Subsequently, multivariate logistic regression analysis was performed. All statistical tests were two-sided, and statistical significance was defined as a *p*-value of <0.05. All statistical analyses were performed using the IBM SPSS Statistics for Windows (version 26.0; IBM Corporation, Armonk, NY, USA).

## 3. Results

### 3.1. Clinical Characteristics

A total of 155 procedures in 151 patients were included in this study. Four patients underwent HIPEC twice due to subsequent recurrence. Details of the patient characteristics are presented in Table 1. The median age of the patients was 55 years (range 16–79), and the median duration of hospitalization was 15 days (range 6–135). The majority of patients had a preoperative American Society of Anesthesiologists (ASA) physical status of one (36.8%) or two (54.8%). Eastern Cooperative Oncology Group (ECOG) performance status was 0 in 133 patients (85.8%), indicating a favorable general condition. The frequent underlying medical conditions were hypertension (27.7%), diabetes mellitus (14.2%), and thromboembolism (7.7%). The patients were categorized in the advanced stage; 56.1% of patients were in stage III and 43.9% of patients were in stage IV. The median PCI score was 8 (range, 0–27). Eighty procedures (51.6%) were performed as IDS, and seventy-one procedures (45.8%) were performed as secondary or higher-order cytoreductive surgeries. CC-0 or CC-1 was achieved in 81.3% of procedures, and 29 procedures were classified as CC-2, with a residual disease diameter of less than 10 mm. The median surgical complexity score was 4 (range 0–12), with 46.5% and 40.6% in low and intermediate complexity groups, respectively, and 12.9% in high complexity groups.

### 3.2. Postoperative Morbidity and Mortality

According to the MSKCC SSE grading system, postoperative morbidity and mortality of any grade occurred in 89.0% of procedures within 30 days after HIPEC (Table 2). Two or more morbidities were reported in 109 procedures (70.3%). The most common morbidities were anemia (79/155, 51.0%), fever (71/155, 45.8%), and pleural effusion (63/155, 40.6%). A total of twenty severe morbidities occurred in eighteen procedures (11.6%), with two procedures simultaneously resulting in two severe morbidities (Table S1). Wound infection was the most common severe morbidity, occurring in five procedures (3.2%). Four patients underwent surgical wound debridement, and one patient required radiologic drainage insertion. Pleural effusion requiring intervention and postoperative hemorrhage were observed in three patients (1.9%).

The incidence of morbidities according to the chemotherapy agents was as follows: In the group using cisplatin, morbidities occurred in the following order: anemia (69.5%), pleural effusion (39.0%), and fever (39.0%), with no serious side effects related to nephrotoxicity. In the paclitaxel group, morbidities occurred in the order of fever (50.0%), pleural effusion (41.7%), and anemia (39.6%). Severe side effects of grade 3 or higher occurred in six procedures with cisplatin and twelve procedures with paclitaxel (Table S2). The incidence of anemia was the only statistically significant difference (*p* < 0.0001), while no differences were found in other morbidities. Although there were no reported cases of acute kidney injury, electrolyte imbalance was observed in a procedure using paclitaxel.

Thirteen patients needed re-hospitalization, and six patients required re-operation due to ≥grade 3 morbidities within 30 days postoperatively (Table S3). The most common reasons for readmission were abdominal pain and ileus. However, most readmitted patients received conservative management, and only two patients required radiologic interventions to resolve the symptoms. Between 31 and 90 days, eight patients were readmitted due to morbidity. Among the patients, two required radiologic interventions due to an infected lymphocele. Postoperative death within 30 days and between 31 and 90 days after surgery was not observed.

The clinical factors associated with severe postoperative morbidities are presented in Table 3. Older age, longer hospitalization, PCI score, bowel resection, CC, and operation time were identified as significant factors associated with an increased likelihood of severe postoperative morbidities. The univariate analysis, employing a logistic regression model, revealed that the PCI score, bowel resection, and operation time were significant risk factors that increased severe postoperative morbidities (Table 4). However, in the multivariate analysis, none of the factors exhibited statistical significance, including bowel resection (OR 2.109; 95% CI, 0.647–6.870, *p* = 0.216).

## 4. Discussion

This retrospective study investigated morbidity and mortality rates following HIPEC for ovarian cancer. Severe morbidity was observed in 11.6% of procedures, and no cases of mortality were reported. The most prevalent severe morbidities were wound infection (3.2%), followed by pleural effusion (1.9%) and postoperative hemorrhage (1.9%). Among morbidities of any grade, anemia (51.0%), fever (45.8%), and pleural effusion (40.6%) were the most frequent (Table 2).

HIPEC necessitated a longer operation time compared to CRS alone due to the circulation of chemotherapy in the abdominal cavity. In addition, owing to the cytotoxic effect of chemotherapy itself, physicians were concerned about postoperative complications after CRS with HIPEC. Previous studies reported severe morbidity rates after CRS in ovarian cancer ranging from 7.4% to 26.2%, whereas the rate for CRS with HIPEC was in the range of 8.6% to 35.7% [6,10,12,13,14,15,20,21,22].

Table 5 shows the summarized results of randomized controlled trials that investigated HIPEC in ovarian cancer, with a focus on morbidity and mortality rates. In a study by van Driel et al., the severe morbidity rate of the IDS with HIPEC arm was 27%, compared to 25% in the IDS-only arm (*p* = 0.76) [6]. Other randomized trials also compared HIPEC and the control in IDS and reported 28.6% morbidity rates in the HIPEC group and 27.8% in the control group without statistical differences [8]. There was also no significant difference in 30-day morbidity in the randomized study of HIPEC in secondary CRS (24% in secondary CRS with HIPEC arm vs. 20% in standard secondary CRS arm) [23]. In a study using paclitaxel as an anticancer drug, the severe morbidity rate was 15.6% [24]. According to previous randomized trials on the treatment of ovarian cancers, the morbidity and mortality rates were not significantly different compared to the control group (Table 5) [6,8,23,24].

In the present study, the severe morbidity rate (12%) was reported to be lower than in the four previously reported randomized trials. This might be a result of several factors. First, the assessment system of adverse events used in this study could affect this result. The MSKCC SSE system or Clavien–Dindo classification that was used in this study is used for grade surgical morbidity. Thus, the grading system was different from the Common Terminology Criteria for Adverse Events (CTCAE), which was used to evaluate adverse events after chemotherapy in some categories. HIPEC combines surgery and chemotherapy and can cause specific adverse events. The differences between grading systems might have underestimated the severe morbidity rate in the present study. Second, as this was a retrospective study, some complications may have been omitted from the investigation. However, even in a retrospective study, the possibility of missing severe morbidities requiring additional intervention seems very low. Other factors that could lower the complication rates than those in previous studies might be advanced surgical techniques and perioperative management.

Chemotherapy drugs could also influence both the incidence rate and morbidity profile. Nephrotoxicity and ototoxicity are major side effects of cisplatin, while neurotoxicity is commonly associated with paclitaxel. In a comparative study on the toxicity profile in HIPEC, the authors compared Mitomycin-C, Oxaliplatin, cisplatin alone, or a combination of cisplatin with Adriamycin [25]. The frequency and profile of severe morbidity differed based on the drug used, with cisplatin showing the lowest side effect rate in that study. In our study, however, there was no significant difference in the occurrence of severe morbidities among the chemotherapy drugs, and there were no reported cases of severe nephrotoxicity with cisplatin. It is possible that the influence of sodium thiosulfate, administrated to prevent nephrotoxicity [26], and the nature of HIPEC, which may result in lower systemic morbidities compared to systemic chemotherapy, could have played a role [27]. Additionally, the pharmacokinetic properties of paclitaxel, originating from its high molecular weight, maintain high concentrations in the peritoneal cavity with lower systemic absorption [28].

Recent studies on HIPEC in ovarian cancers reported a mortality rate of 0–6.3% (Table 5). In previous studies on non-gynecologic cancers, morbidity and mortality rates have been reported to be higher than those of gynecologic cancers. According to a systematic review and randomized trial of HIPEC in colorectal cancer, the major morbidity range was reported as 15.1–47.2%, and mortality was 0–4.5% [29,30]. In a retrospective multicenter study, postoperative mortality was 4.1–6.5% in non-gynecologic cancers [31,32]. In contrast, in a large retrospective study by Bakrin et al., the mortality rate was reported as 0.8% in ovarian cancers, and several prospective studies reported a 0–1% of mortality rate [6,12,33]. The lower mortality rate of gynecological cancers could be related to fewer bowel surgeries in ovarian cancers than in gastrointestinal cancers [34,35].

Based on the univariate logistic analysis, the PCI score, bowel resection, and operation time were possible risk factors related to the occurrence of severe morbidity in our study. However, there were no statistically significant risk factors in the multivariate analysis. In retrospective study of Bakrin et al., a PCI score > 8 was related to the occurrence of postoperative morbidity (OR 2.17, *p* = 0.003) after HIPEC in patients with ovarian cancer. Other contributing factors on postoperative morbidity were HIPEC as the first-line treatment (OR 1.7, *p* = 0.008), CC-1 or CC-2 (OR 2.06, *p* = 0.031), and the use of cisplatin (OR 3.08, *p* = 0.002) [12]. In another retrospective study that dealt with postoperative mortality and morbidity, the number of intraoperative blood transfusions and the PCI score were predictors of severe postoperative morbidity. However, a strong association between morbidity and related factors was not revealed; the odds ratios were 1.17 and 1.04 for transfusions and the PCI score, respectively [36].

The risk factors for postoperative morbidity were similar in studies on CRS in ovarian cancers, such as performance status, age, albumin level, and complexity or extensive surgery [18,21,37]. PDS showed a higher morbidity rate than IDS with neoadjuvant chemotherapy in previous studies [20,21,38]. However, in our study, the timing of HIPEC did not significantly impact the morbidity rate. The majority of the patients underwent IDS or surgery after recurrence, while only 2.6% (4/155) underwent HIPEC for the PDS. Due to the limited sample size, a statistically significant difference was not observed. In addition, the composition of our study population may be related to lower surgical complexity in our study, which may lead to a lower severe morbidity rate. Considering that studies dealing with HIPEC and CRS suggest similar risk factors, it was presumed that extensive surgery and the patient’s general condition affect the incidence of morbidity or mortality after surgery.

As a retrospective study, this study had several limitations. For data collection on postoperative morbidity, medical records were relied upon, introducing the possibility of selection bias. The choice of the morbidity grading system may have influenced the study outcomes. However, it is important to note that the morbidities were well-managed with medical treatment, and we maintained data consistency by applying a single grading system. In addition, as a relatively small study, the number of bowel resections or infrequent surgical procedures, such as pancreatectomy and splenectomy, were not included in the statistical analyses but may be related to the incidence of morbidity.

The significance of this study lies in confirming the safety of HIPEC in the real world and exploring the risk factors associated with postoperative morbidity, which may help in establishing strategies to reduce complications. Since postoperative morbidity could be related to poor oncologic outcomes [13,39,40], proper patient selection based on possible risk factors could reduce the morbidity rate and improve oncologic outcomes. Based on these results, further research is needed to achieve better clinical outcomes.

## 5. Conclusions

In this study, the rate of severe morbidity was reported to be 11.4%, and the PCI score, bowel resection, and operation time had statistically significant relationships with severe morbidity in the univariate analysis. However, no significant risk factors were found in the multivariate analysis. However, the overall morbidity and mortality rates were relatively low and acceptable compared to those reported in previous studies. In real-world settings, the severe morbidity rate was not significantly affected by factors such as the timing of HIPEC, IDS, recurrence, or the choice of chemotherapy agent. With proper patient selection and perioperative management, HIPEC could be a safe and better treatment option for ovarian cancer patients with peritoneal carcinomatosis.

## Figures and Tables

**Table 1:** Clinical characteristics of patients.

		Number of Procedures = 155
Age (years), median (range)		55 (16–79)
BMI (kg/m^2^), median (range)		23.1 (15.2–34.5)
Length of stay (days), median (range)		15 (6–135)
ASA class	1	57 (36.8%)
	2	85 (54.8%)
	3	13 (8.4%)
ECOG performance status	0	133 (85.8%)
	1	22 (14.2%)
Underlying disease	HTN	43 (27.7%)
	DM	22 (14.2%)
	Thromboembolism	12 (7.7%)
FIGO stage	III	87 (56.1%)
	IV	68 (43.9%)
Origin	Ovary	138 (89.0%)
	Fallopian tube	3 (1.9%)
	Peritoneum	14 (9.0%)
Histologic type	High grade serous	124 (80%)
	Mucinous	8 (5.2%)
	Clear cell	8 (5.2%)
	Low grade serous	4 (2.6%)
	Endometrioid	1 (0.6%)
	Other *	10 (6.5%)
PCI score, median (range)		8 (0–27)
Timing of Surgery	Primary debulking surgery	4 (2.6%)
	Interval debulking surgery	80 (47.0%)
	≥2nd debulking surgery	71 (45.8%)
Chemotherapy agents	Paclitaxel	96 (61.9%)
	Cisplatin	59 (38.1%)
CC	CC-0	112 (72.3%)
	CC-1	14 (9.0%)
	CC-2	29 (18.7%)
Administration method	Open	146 (94.2%)
	Close	9 (5.8%)
SCS, median(range)		4 (0–12)
SCS group	Low (<3)	72 (46.5%)
	Intermediate (4–7)	63 (40.6%)
	High (>8)	20 (12.9%)
EBL (mL), median (range)		580 (10–8600)
OP time (min), median (range)		470 (195–1080)

BMI, body mass index; ASA, American Society of Anesthesiologists physical status classification; ECOG, Eastern Cooperative Oncology Group; DM, diabetes mellitus; HTN, hypertension; FIGO, International Federation of Gynecology and Obstetrics; PCI, peritoneal carcinomatosis index; CC, completeness of cytoreduction; SCS, surgical complexity score; EBL, estimated blood loss; OP, operation. * carcinosarcoma, granulosa cell tumor, mesothelioma of ovary, mesonephric adenocarcinoma, sero-mucinous carcinoma, Sertoli–Leydig cell tumor, and undifferentiated carcinoma.

**Table 2:** Detailed information of morbidity and mortality after HIPEC within 30 days.

Organ System		Any Grade (%)	Grade 3–5
		(Total Procedure = 155)	
General	Poor oral intake/general weakness	1 (0.6%)	0
Cardiovascular system	Hypotension	2 (1.3%)	0
	Arrhythmia	4 (2.6%)	0
Head and neck	Salivary gland infection	1 (0.6%)	0
Gastrointestinal system	Ileus	34 (21.9%)	0
	Nausea/Vomiting	1 (0.6%)	0
	Gastrointestinal bleeding	1 (0.6%)	0
	Anastomotic stricture	1 (0.6%)	1 (0.6%)
	Constipation	1 (0.6%)	0
	Pancreatitis	1 (0.6%)	0
	Small bowel obstruction	1 (0.6%)	0
	Small bowel perforation	1 (0.6%)	1 (0.6%)
	Non infective intra-abdominal fluid collection	2 (1.3%)	2 (1.3%)
	Chylous ascites	2 (1.3%)	0
	Liver enzyme elevation	3 (1.9%)	0
	Lipase elevation	1 (0.6%)	0
Pulmonary system	Pleural effusion	63 (40.6%)	3 (1.9%)
	Pneumonia	2 (1.3%)	0
	Atelectasis	2 (1.3%)	0
	Pneumothorax	2 (1.3%)	0
	Hypoxia	1 (0.6%)	0
	Pulmonary edema	1 (0.6%)	0
Genitourinary system	Urinary retention	6 (3.9%)	0
	Hydronephrosis	1 (0.6%)	0
	Ureter stricture and fistula	1 (0.6%)	1 (0.6%)
Metabolic	Electrolyte imbalance	1 (0.6%)	0
Hematologic or Vascular system	Anemia	79 (51.0%)	0
	Thrombocytopenia	17 (11.0%)	0
	Neutropenia	19 (12.3%)	0
	Febrile neutropenia	1 (0.6%)	0
	Deep vein thrombosis	1 (0.6%)	0
	Pulmonary embolism	3 (1.9%)	1 (0.6%)
	Disseminated intravascular coagulation	1 (0.6%)	0
	Postoperative hemorrhage	3 (1.9%)	3 (1.9%)
	Hematoma	1 (0.6%)	0
Musculoskeletal system	Lymphedema	1 (0.6%)	0
Nervous system	Delirium	9 (5.8%)	0
	Vocal cord paralysis	1 (0.6%)	0
Pain	Abdominal pain	5 (3.2%)	0
Wound or skin	Wound dehiscence	10 (6.5%)	0
	Sore	1 (0.6%)	0
Infection	Fever	71 (45.8%)	0
	Wound infection	7 (4.5%)	5
	Catheter related infection	2 (1.3%)	2 (1.3%)
	Urinary tract infection	1 (0.6%)	0
	Intra-abdominal infection	1 (0.6%)	1 (0.6%)

**Table 3:** Clinical factors associated with postoperative severe morbidity.

		≤Grade 2 AE (137 Procedures)	≥Grade 3 AE (18 Procedures)	*p*-Value
Age (year), median (range)		54 (16–79)	61 (32–70)	0.026
Length of stay (day), median (range)		14 (6–46)	26 (14–135)	<0.001
Underlying disease	HTN	36 (26.3%)	7 (38.9%)	0.272
	DM	14 (13.1%)	4 (22.2%)	0.291
	Thromboembolism	11 (7.4%)	1 (5.6%)	1.000
ASA class	1	49 (35.8%)	8 (44.4%)	0.236
	2	75 (54.7%)	10 (55.6%)	
	3	13 (9.5%)	0 (0%)	
PCI, median (range)		8 (0–25)	12 (2–27)	0.013
Timing of Surgery	Primary debulking surgery	4 (2.9%)	0 (0%)	0.920
	Interval debulking surgery	70 (51.1%)	10 (55.6%)	
	≥2nd debulking surgery	63 (46.0%)	8 (44.4%)	
Operation extent	Hysterectomy	68 (49.6%)	9 (50.0%)	0.977
	Lymph node dissection	59 (43.1%)	7 (38.9%)	0.736
	Peritonectomy	84 (61.3%)	13 (72.2%)	0.369
	Bowel resection	25 (18.2%)	8 (44.4%)	0.027
	Hepatectomy	23 (16.8%)	1 (5.6%)	0.311
	Diaphragmatic stripping	40 (29.2%)	7 (38.9%)	0.400
	Splenectomy	17 (12.4)	3 (16.7%)	0.706
SCS	Low	66 (48.2%)	6 (28.6%)	0.463
	Intermediate	53 (38.7%)	10 (55.6%)	
	High	18 (13.1%)	2 (11.1%)	
CC	0	103 (75.2%)	9 (50.0%)	0.016
	1	12 (8.8%)	2 (11.1%)	
	2	22 (16.1%)	7 (38.9%)	
Chemotherapy agents	Paclitaxel	84 (56.4%)	12 (57.1%)	0.660
	Cisplatin	53 (35.6%)	6 (28.6%)	
OP time (min), median (range)		460 (195–1080)	557.5 (400–1080)	0.007
EBL (mL), median (range)		550 (10–8600)	800 (200–5050)	0.224

AE, adverse event; HTN, hypertension; DM, diabetes mellitus; PCI, peritoneal carcinomatosis index; SCS, surgical complexity score; CC, completeness of cytoreduction; OP, operation; EBL, estimated blood loss.

**Table 4:** Univariate and multivariate analysis of risk factors associated with occurrence of severe morbidity.

	Univariate			Multivariate		
	OR	95% CI	*p*-Value	OR	95% CI	*p*-Value
Age	1.049	0.995–1.106	0.075			
PCI score	1.106	1.022–1.196	0.012	1.066	0.971–1.170	0.182
Bowel Resection	3.584	1.285–9.997	0.015	2.109	0.647–6.870	0.216
OP time	1.003	1.001–1.006	0.011	1.002	0.998–1.005	0.385
CC			0.066			
CC-1	1.907	0.368–9.879				
CC-2	3.641	1.225–10.828				

OR, odds ratio; CI, confidence interval; PCI, peritoneal carcinomatosis index; OP, operation; CC, completeness of cytoreduction.

**Table 5:** Previously reported randomized studies of HIPEC in gynecologic cancers.

	van Driel et al. (2018) [6]	Zivanovic et al. (2021) [23]	Antonio et al. (2022) [8]	Campos et al. (2024) [24]	Present Study
N of HIPEC arm	122	49	35	32	155
Age (years), median (range)	61 (IQR, 55–66)	59 (39–74)	56 (29–75)	60.34 (±11.7)	55 (16–79)
PCI, median(range)	-	-	10 (2–22)	-	8 (0–27)
Complete resection (%)	84 (69%)	40 (82%)	33 (94.3%)	32 (100%)	112 (72.3%)
Timing of surgery	IDS	2nd CRS	IDS	PDS, IDS, 2nd CRS	PDS, IDS, ≥2nd CRS
Anticancer drug	Cisplatin	Carboplatin	Cisplatin	Paclitaxel	Paclitaxel, Cisplatin
AE assessment system	CTCAE ver 4.0	MSKCC SSE	CTCAE ver 3.0	Cavien-Dindo	MSKCC SSE
≥Grade 3 AE (%)	32 (27%)	12 (24%)	10 (28.6%)	5 (15.6%)	18 (11.6%)
Most common AE, Grade 3–5 (%)	Abdominal pain (6%), Infection (5%), Ileus (4%)	-	Anemia (11.4%), Ileus (5.7%)	-	Wound infection (3.2%) Pleural effusion (1.9%) Postoperative hemorrhage (1.9%)
Mortality rate (%)	0	0	1 (2.8%)	2 (6.3%)	0

HIPEC, hyperthermic intraperitoneal chemotherapy; IQR, Interquartile range; PCI, peritoneal carcinomatosis index; IDS, interval debulking surgery; CRS, cytoreductive surgery; PDS, primary debulking surgery; AE, adverse event; CTCAE, Common Terminology Criteria for Adverse Events; MSKCC SSE, Memorial Sloan Kettering Cancer Center Surgical Secondary Events System.

## Data Availability

The participants of this study did not give written consent for their data to be shared publicly; so, due to the sensitive nature of the research, supporting data are not available.

## Supplementary Materials

The following supporting information can be downloaded at: https://www.mdpi.com/article/10.3390/jcm14051782/s1, Table S1: Severe adverse events after HIPEC within 30 days; Table S2: Detailed morbidity profile according to the chemotherapy agent; Table S3: The causes of readmission and reoperation within 30 days and 31 to 90 days.

## Abbreviations

The following abbreviations are used in this manuscript:

HIPEC	Hyperthermic intraperitoneal chemotherapy
CRS	Cytoreductive surgery
IP	Intraperitoneal
IDS	Interval debulking surgery
PDS	Primary debulking surgery
PCI	Peritoneal carcinomatosis index
CC	Completeness of the cytoreduction
MSKCC	The Memorial Sloan Kettering Cancer Center
SSE	Surgical secondary event
ASA	American society of Anesthesiologists
ECOG	Eastern Cooperative oncology Group
CTCAE	Common Terminology Criteria for Adverse Events
SCS	Surgical complexity score

## References

[1] Burg L., Timmermans M., van der Aa M., Boll D., Rovers K., de Hingh I., van Altena A. (2020). Incidence and predictors of peritoneal metastases of gynecological origin: A population-based study in the Netherlands. J. Gynecol. Oncol..

[2] Berek J.S., Renz M., Kehoe S., Kumar L., Friedlander M. (2021). Cancer of the ovary, fallopian tube, and peritoneum: 2021 update. Int. J. Gynaecol. Obstet..

[3] Armstrong D.K., Bundy B., Wenzel L., Huang H.Q., Baergen R., Lele S., Copeland L.J., Walker J.L., Burger R.A. (2006). Intraperitoneal cisplatin and paclitaxel in ovarian cancer. N. Engl. J. Med..

[4] Jaaback K., Johnson N., Lawrie T.A. (2011). Intraperitoneal chemotherapy for the initial management of primary epithelial ovarian cancer. Cochrane Database Syst. Rev..

[5] Hess L.M., Benham-Hutchins M., Herzog T.J., Hsu C.H., Malone D.C., Skrepnek G.H., Slack M.K., Alberts D.S. (2007). A meta-analysis of the efficacy of intraperitoneal cisplatin for the front-line treatment of ovarian cancer. Int. J. Gynecol. Cancer.

[6] van Driel W.J., Koole S.N., Sikorska K., van Leeuwen J.H.S., Schreuder H.W.R., Hermans R.H.M., de Hingh I.H.J.T., van der Velden J., Arts H.J., Massuger L.F.A.G. (2018). Hyperthermic Intraperitoneal Chemotherapy in Ovarian Cancer. N. Engl. J. Med..

[7] Lim M.C., Chang S.-J., Park B., Yoo H.J., Yoo C.W., Nam B.H., Park S.-Y. (2022). Survival After Hyperthermic Intraperitoneal Chemotherapy and Primary or Interval Cytoreductive Surgery in Ovarian Cancer: A Randomized Clinical Trial. JAMA Surg..

[8] Antonio C.C.P., Gil Alida G., Elena G.G., Rocío G.S., Jerónimo M.G., Luis A.R.J., Aníbal N.D., Francisco B.V., Jesús G.R.Á., Pablo R.R. (2022). Cytoreductive Surgery With or Without HIPEC After Neoadjuvant Chemotherapy in Ovarian Cancer: A Phase 3 Clinical Trial. Ann. Surg. Oncol..

[9] Charo L.M., Jou J., Binder P., Hohmann S.F., Saenz C., McHale M., Eskander R.N., Plaxe S. (2020). Current status of hyperthermic intraperitoneal chemotherapy (HIPEC) for ovarian cancer in the United States. Gynecol. Oncol..

[10] Zivanovic O., Chi D.S., Filippova O., Randall L.M., Bristow R.E., O’Cearbhaill R.E. (2018). It’s time to warm up to hyperthermic intraperitoneal chemotherapy for patients with ovarian cancer. Gynecol. Oncol..

[11] Gagnière J., Veziant J., Pereira B., Pezet D., Le Roy B., Slim K. (2018). Cytoreductive Surgery and Hyperthermic Intraperitoneal Chemotherapy for the Elderly: Is It Reasonable? A Meta-Analysis. Ann. Surg. Oncol..

[12] Bakrin N., Bereder J., Decullier E., Classe J., Msika S., Lorimier G., Abboud K., Meeus P., Ferron G., Quenet F. (2013). Peritoneal carcinomatosis treated with cytoreductive surgery and Hyperthermic Intraperitoneal Chemotherapy (HIPEC) for advanced ovarian carcinoma: A French multicentre retrospective cohort study of 566 patients. Eur. J. Surg. Oncol..

[13] Muñoz-Casares F., Medina-Fernández F., Arjona-Sánchez Á., Casado-Adam Á., Sánchez-Hidalgo J., Rubio M., Ortega-Salas R., Muñoz-Villanueva M., Rufián-Peña S., Briceño F. (2016). Peritonectomy procedures and HIPEC in the treatment of peritoneal carcinomatosis from ovarian cancer: Long-term outcomes and perspectives from a high-volume center. Eur. J. Surg. Oncol..

[14] Lee Y.J., Lee J.-Y., Cho M.-S., Nam E.J., Kim S.W., Kim S., Kim Y.T. (2019). Incorporation of paclitaxel-based hyperthermic intraperitoneal chemotherapy in patients with advanced-stage ovarian cancer treated with neoadjuvant chemotherapy followed by interval debulking surgery: A protocol-based pilot study. J. Gynecol. Oncol..

[15] Lee Y.J., Seon K.E., Jung D.C., Lee J.-Y., Nam E.J., Kim S.W., Kim S., Kim Y.T. (2022). Interval debulking surgery with or without hyperthermic intraperitoneal chemotherapy in advanced-stage ovarian cancer: Single-institution cohort study. Front. Oncol..

[16] Jacquet P., Sugarbaker P.H. (1996). Clinical research methodologies in diagnosis and staging of patients with peritoneal carcinomatosis. Cancer Treat. Res..

[17] Aletti G., Dowdy S.C., Podratz K.C., Cliby W.A. (2007). Relationship among surgical complexity, short-term morbidity, and overall survival in primary surgery for advanced ovarian cancer. Am. J. Obstet. Gynecol..

[18] Aletti G., Dowdy S.C., Podratz K.C., Cliby W.A. (2007). A new frontier for quality of care in gynecologic oncology surgery: Multi-institutional assessment of short-term outcomes for ovarian cancer using a risk-adjusted model. Gynecol. Oncol..

[19] Strong V.E., Selby L.V., Sovel M., Disa J.J., Hoskins W., Dematteo R., Scardino P., Jaques D.P. (2015). Development and assessment of Memorial Sloan Kettering Cancer Center’s Surgical Secondary Events grading system. Ann. Surg. Oncol..

[20] Machida H., Tokunaga H., Matsuo K., Matsumura N., Kobayashi Y., Tabata T., Kaneuchi M., Nagase S., Mikami M. (2020). Survival outcome and perioperative complication related to neoadjuvant chemotherapy with carboplatin and paclitaxel for advanced ovarian cancer: A systematic review and meta-analysis. Eur. J. Surg. Oncol..

[21] Kengsakul M., Boer G.M.N.-D., Udomkarnjananun S., Kerr S.J., Niehot C.D., van Beekhuizen H.J. (2022). Factors predicting postoperative morbidity after cytoreductive surgery for ovarian cancer: A systematic review and meta-analysis. J. Gynecol. Oncol..

[22] Norppa N., Staff S., Helminen M., Auranen A., Saarelainen S. (2022). Improved survival after implementation of ultra-radical surgery in advanced epithelial ovarian cancer: Results from a tertiary referral center. Gynecol. Oncol..

[23] Zivanovic O., Chi D.S., Zhou Q., Iasonos A., Konner J.A., Makker V., Grisham R.N., Brown A.K., Nerenstone S., Diaz J.P. (2021). Secondary Cytoreduction and Carboplatin Hyperthermic Intraperitoneal Chemotherapy for Platinum-Sensitive Recurrent Ovarian Cancer: An MSK Team Ovary Phase II Study. J. Clin. Oncol..

[24] Campos P.V., García S.S., Amo-Salas M., Santos E.G., de la Manzanara C.L., Alberca A., Padilla-Valverde D., Calvo F.J.R., Martín J. (2024). Paclitaxel as HIPEC-Drug after Surgical Cytoreduction for Ovarian Peritoneal Metastases: A Randomized Phase III Clinical Trial (HIPECOVA). Curr. Oncol..

[25] Somashekhar S., Yethadka R., Kumar C.R., Ashwin K., Zaveri S., Rauthan A. (2020). Toxicity profile of chemotherapy agents used in cytoreductive surgery and hyperthermic intraperitoneal chemotherapy for peritoneal surface malignancies. Eur. J. Surg. Oncol..

[26] Laplace N., Kepenekian V., Friggeri A., Vassal O., Ranchon F., Rioufol C., Gertych W., Villeneuve L., Glehen O., Bakrin N. (2020). Sodium thiosulfate protects from renal impairement following hyperthermic intraperitoneal chemotherapy (HIPEC) with Cisplatin. Int. J. Hyperth..

[27] Lim P.-Q., Han I.-H., Seow K.-M., Chen K.-H. (2022). Hyperthermic Intraperitoneal Chemotherapy (HIPEC): An Overview of the Molecular and Cellular Mechanisms of Actions and Effects on Epithelial Ovarian Cancers. Int. J. Mol. Sci..

[28] Sugarbaker P.H. (2021). Intraperitoneal paclitaxel: Pharmacology, clinical results and future prospects. J. Gastrointest. Oncol..

[29] Flood M., Narasimhan V., Waters P., Ramsay R., Michael M., Warrier S., Heriot A. (2021). Survival after cytoreductive surgery and hyperthermic intraperitoneal chemotherapy for colorectal peritoneal metastases: A systematic review and discussion of latest controversies. Surgeon.

[30] Quénet F., Elias D., Roca L., Goéré D., Ghouti L., Pocard M., Facy O., Arvieux C., Lorimier G., Pezet D. (2021). Cytoreductive surgery plus hyperthermic intraperitoneal chemotherapy versus cytoreductive surgery alone for colorectal peritoneal metastases (PRODIGE 7): A multicentre, randomised, open-label, phase 3 trial. Lancet Oncol..

[31] de Chirurgie A.F., Glehen O., Gilly F.N., Arvieux C., Cotte E., Boutitie F., Mansvelt B., Bereder J.M., Lorimier G., Quenet F. (2010). Peritoneal carcinomatosis from gastric cancer: A multi-institutional study of 159 patients treated by cytoreductive surgery combined with perioperative intraperitoneal chemotherapy. Ann. Surg. Oncol..

[32] Glehen O., Gilly F.N., Boutitie F., Bereder J.M., Quenet F., Sideris L., Mansvelt B., Lorimier G., Msika S., Elias D. (2010). Toward curative treatment of peritoneal carcinomatosis from nonovarian origin by cytoreductive surgery combined with perioperative intraperitoneal chemotherapy: A multi-institutional study of 1290 patients. Cancer.

[33] Lim M.C., Kang S., Choi J., Song Y.J., Park S., Seo S.-S., Park S.-Y. (2009). Hyperthermic intraperitoneal chemotherapy after extensive cytoreductive surgery in patients with primary advanced epithelial ovarian cancer: Interim analysis of a phase II study. Ann. Surg. Oncol..

[34] Newton A.D., Bartlett E.K., Karakousis G.C. (2016). Cytoreductive surgery and hyperthermic intraperitoneal chemotherapy: A review of factors contributing to morbidity and mortality. J. Gastrointest. Oncol..

[35] Franko J., Gusani N.J., Holtzman M.P., Ahrendt S.A., Jones H.L., Zeh H.J., Bartlett D.L. (2008). Multivisceral resection does not affect morbidity and survival after cytoreductive surgery and chemoperfusion for carcinomatosis from colorectal cancer. Ann. Surg. Oncol..

[36] Macrì A., Accarpio F., Arcoraci V., Casella F., De Cian F., De Iaco P., Orsenigo E., Roviello F., Scambia G., Saladino E. (2021). Predictors of morbidity and mortality in patients submitted to cytoreductive surgery plus hyperthermic intraperitoneal chemotherapy for ovarian carcinomatosis: A multicenter study. Pleura Peritoneum.

[37] Cham S., Chen L., Clair C.M.S., Hou J.Y., Tergas A.I., Melamed A., Ananth C.V., Neugut A.I., Hershman D.L., Wright J.D. (2019). Development and validation of a risk-calculator for adverse perioperative outcomes for women with ovarian cancer. Am. J. Obstet. Gynecol..

[38] Fagotti A., Ferrandina G., Vizzielli G., Fanfani F., Gallotta V., Chiantera V., Costantini B., Margariti P.A., Alletti S.G., Cosentino F. (2016). Phase III randomised clinical trial comparing primary surgery versus neoadjuvant chemotherapy in advanced epithelial ovarian cancer with high tumour load (SCORPION trial): Final analysis of peri-operative outcome. Eur. J. Cancer.

[39] Schneider M.A., Eshmuminov D., Lehmann K. (2017). Major Postoperative Complications Are a Risk Factor for Impaired Survival after CRS/HIPEC. Ann. Surg. Oncol..

[40] Simkens G.A., van Oudheusden T.R., Luyer M.D., Nienhuijs S.W., Nieuwenhuijzen G.A., Rutten H.J., de Hingh I.H. (2015). Serious Postoperative Complications Affect Early Recurrence After Cytoreductive Surgery and HIPEC for Colorectal Peritoneal Carcinomatosis. Ann. Surg. Oncol..

