# Supplementary material for: Morbidity and Mortality Outcomes After Cytoreductive Surgery with Hyperthermic Intraperitoneal Chemotherapy for Treatment of Ovarian Cancer
Source: J Clin Med. 2025 Mar 6;14(5):1782. doi: 10.3390/jcm14051782 (PMC11901296; doi:10.3390/jcm14051782)
Supplement: Supplementary file 1 [file jcm-14-01782-s001.zip › jcm-3464216-supplementary.pdf]

Table S1. Severe adverse events after HIPEC within 30 days

	Grade 3/4 (Total procedure=155)
Wound infection	5 (3.2%)
Pleural effusion	3 (1.9%)
Postoperative Hemorrhage	3 (1.9%)
Catheter related infection*	2 (1.3%)
Non-infective intra-abdominal fluid collection	2 (1.3%)
Pulmonary embolism	1 (0.6%)
Intra-abdominal infection	1 (0.6%)
Anastomotic stricture	1 (0.6%)
Small bowel perforation	1 (0.6%)
Vesicovaginal fistula, ureter stent displace	1 (0.6%)

* Chemoport related infection (n=1), Surgical drainage catheter infection (n=1)

Table S2. Detailed morbidity profile accorrding to the chemotherapy agent

Organ system		Cisplatin (n = 59)		Paclitaxel (n = 96)	
		Any grade	Grade 3-5	Any grade	Grade 3-5
General	Poor oral intake/general weakness	0	0	1 (1.0%)	0
Cardiovascular system	Hypotension	0	0	2 (2.1%)	0
	Arrhythmia	0	0	3 (3.1%)	0
Head and neck	Salivary gland infection	0	0	1 (1.0%)	0
Gastrointestinal system	Ileus	12 (20.3%)	0	22 (22.9%)	0
	Nausea/Vomiting	0	0	1 (1.0%)	0
	Gastrointestinal bleeding	0	0	1 (1.0%)	0
	Anastomotic stricture	0	0	0	1 (1.0%)
	Constipation	0	0	1 (1.0%)	0
	Pancreatitis	1 (1.7%)	0	0	0
	Small bowel obstruction	1 (1.7%)	0	0	0
	Small bowel perforation	1 (1.7%)	1 (1.7%)	0	0
	Non infective intra-abdominal fluid collection	0	2 (3.4%)	0	0
	Chylous ascites	0	0	2 (2.1%)	0
	Liver enzyme elevation	0	0	3 (3.1%)	0
	Lipase elevation	1 (1.7%)	0	0	0
Pulmonary system	Pleural effusion	23 (39.0%)	1 (1.7%)	40 (41.7%)	2 (2.1%)
	Pneumonia	1 (1.7%)	0	1 (1.0%)	0
	Atelectasis	0	0	2 (2.1%)	0
	Pneumothorax	2 (3.4%)	0	0	0
	Hypoxia	0	0	1 (1.0%)	0
	Pulmonary edema	0	0	1 (1.0%)	0
Genitourinary system	Urinary retention	4 (6.8%)	0	2 (2.1%)	0
	Hydronephrosis	0	0	1 (1.0%)	0
	Ureter stricture & fistula	0	0	0	1 (1.0%)
Metabolic	Electrolyte imbalance	0	0	1 (1.0%)	0

Hematologic or Vascular system	Anemia	41 (69.5%)	0	38 (39.6%)	0
	Thrombocytopenia	4 (6.8%)	0	13 (13.5%)	0
	Neutropenia	7 (11.9%)	0	12 (12.5%)	0
	Febrile neutropenia	0	0	1 (1.0%)	0
	Deep vein thrombosis	1 (1.7%)	0	0	0
	Pulmonary embolism	2 (3.4%)	1 (1.7%)	1 (1.0%)	0
	Disseminated intravascular coagulation	0	0	1 (1.0%)	0
	Postoperative hemorrhage	1 (1.7%)	1 (1.7%)	2 (2.1%)	2 (2.1%)
	Hematoma	0	0	1 (1.0%)	0
Musculoskeletal system	Lymphedema	1 (1.7%)	0	0	0
Nervous system	Delirium	7 (11.9%)	0	2 (2.1%)	0
	Vocal cord paralysis	0	0	1 (1.0%)	0
Pain	Abdominal pain	2 (3.4%)	0	3 (3.1%)	0
Wound or skin	Wound dehiscence	5 (8.5%)	0	5 (5.2%)	0
	Sore	0	0	1 (1.0%)	0
Infection	Fever	23 (39.0%)	0	48 (50.0%)	0
	Wound infection	1 (1.7%)	0	6 (6.3%)	5 (5.2%)
	Catheter related infection	0	1 (1.7%)	0	1 (1.0%)
	Urinary tract infection	0	0	1 (1.0%)	0
	Intra-abdominal infection	0	0	0	1 (1.0%)

Table S3. The causes of readmission & reoperation within 30 days and 31 to 90 days

Readmission	Within 30 days (Total procedure=155)	31- to 90-days (Total procedure=155)
Abdominal pain	3 (1.9%)	0
Ileus	3 (1.9%)	3 (1.9%)
Wound infection	1 (0.6%)	1 (0.6%)
Poor oral intake	1 (0.6%)	1 (0.6%)
Vesicovaginal fistula, ureter stent displace*	1 (0.6%)	0
Catheter related infection*	1 (0.6%)	0
General weakness	1 (0.6%)	0
Constipation	1 (0.6%)	0
Fever	1 (0.6%)	0
Infected lymphocele*	0	2 (1.3%)
Small bowel obstruction	0	1 (0.6%)
Reoperation		
Wound infection	4 (2.6%)	1 (0.6%)
Small bowel perforation	1 (0.6%)	0
Postoperative hemorrhage	1 (0.6%)	0

* Required radiologic interventions
